# Chirality-assisted enhancement of tripartite entanglement in waveguide QED

**DOI:** 10.1038/s41598-024-61043-0

**Published:** 2024-05-15

**Authors:** Logan Patrick, Umar Arshad, Dingyu Guo, Imran Mirza

**Affiliations:** Macklin Quantum Information Sciences, Department of Physics, Miami University, Oxford, Ohio 45056 USA

**Keywords:** Waveguide QED, Multipartite entanglement, Chiral quantum optics, Single photons and quantum effects, Qubits, Quantum information

## Abstract

We study the generation and control of genuine tripartite entanglement among quantum emitters (QEs) that are side-coupled to one-dimensional spin-momentum locked (or chiral) waveguides. By applying the machinery of Fock state master equations along with the recently proposed concurrence fill measure of tripartite entanglement [S. Xie and J. H. Eberly, Phys. Rev. Lett. 127, 040403 (2021)], we analyze how three-photon Gaussian wavepackets can distribute entanglement among two and three QEs. We show that with a five times larger waveguide decay rate in the right direction as compared to the left direction, the maximum value of tripartite entanglement can be elevated by $35\%$ as compared to the symmetric scenario where both left, and right direction decay rates are equal. Additionally, chirality can maintain the tripartite entanglement for longer than the corresponding symmetric decay rate. Finally, we study the influence of detunings and spontaneous emission on the resulting entanglement. We envision quantum networking and long-distance quantum communication as two main areas of applications of this work.

## Introduction

Quantum entanglement, an information resource, lies at the heart of several quantum-enabled technologies^1,2^. However, when exposed to environmental interactions, entanglement is known to behave in a pretty fragile manner, leading to known phenomena of entanglement sudden death^3^ and early-stage disentanglement^4,5^. Thus, one of the main challenges in developing quantum information technologies is to devise ways to sustain entanglement for a long enough time so that the information protocol can be reliably completed. Due to the advancement in the manufacturing of optical elements and atomic trapping techniques, waveguide quantum electrodynamics (wQED) has gained a lot of interest in recent years^6^. Furthermore, the design of the wQED platforms makes them a promising candidate for quantum information processing tasks in general and long-distance quantum communications & quantum networking protocols, in particular^7,8^.

In addition to achieving strong-light matter interaction (typically one of the essential requirements for entanglement generation), in more recent years, direction-dependent preferential photon emission/absorption (also known as chirality) has been demonstrated in wQED architectures^9^. Hence, in the last five years or so, chiral quantum optics has been achieved in various physical platforms^10–12^ and the area has witnessed a variety of novel effects^13–18^. For instance, we have studied emitter-emitter entanglement dynamics in chiral and non-chiral wQED^19–21^. In particular, for the case of single and two photons, we have shown that for strongly coupled wQED case, such chiral light-matter interaction can lead to enhancement in the maximum value of bipartite emitter-emitter entanglement by a factor of 3/2 and 2, respectively, as compared to the corresponding non-chiral coupling case^22,23^.

The need to establish and sustain entanglement among distant nodes of a quantum network for multiple users requires one to go beyond the bipartite entanglement and enter the domain of multiparty entanglement^24,25^. The multipartite entanglement offers new types of applications in quantum computation, for instance, in the context of cluster states and measurement-based computing^26–28^. Keeping in view these essential applications in quantum informatics, in this work, we study three-photon induced qubit-qubit entanglement in wQED. In particular, we pay attention to how the chiral light-matter interaction aids in accomplishing higher values of genuine tripartite entanglement and longer survival times. Worthwhile to emphasize here is the fact that the discussion of the tripartite entanglement should not be treated as a straightforward extension of single or two-photon entanglement problems, but by doing so, we enter the more challenging domain of multipartite entanglement^29,30^ where tripartite entanglement can serve as the first case study.

As the theoretical tools, we work within the framework of Fock state master equations^31–33^ and calculate the genuine tripartite entanglement among up to three two-level QEs using concurrence and concurrence fill criteria^34^.

A literature survey on entanglement in wQED reveals that Zheng et al. have studied entanglement generation between two spatially separated qubits/QEs coupled to a bidirectional waveguide in 2013^35^. They reported steady-state entanglement (quantified through concurrence) $\sim 40\%$ in their work. C.G-Ballestro et al., in Ref.^36^, have concluded $20- 30\%$ entanglement between two qubits in the wQED setting, which was induced by two photons that were launched from the opposite ends of the waveguide. In Ref.^37^, Liao et al. have shown the maximum entanglement of $\sim 40\%$ is attainable if the two qubits are separated by $0.125\,\lambda _0$ with $\lambda _0$ being the resonant wavelength in their study. On the other hand, Mirza et al. (one of the authors of the present manuscript) have reported that entanglement of $\sim 70\%$ can be reached between two qubits using chiral light-matter interfaces when a single photon Gaussian wavepacket induces the entanglement^22^. In this work, by entering the multiphoton processes, as some of the main findings, we find that, as compared to the corresponding non-chiral (symmetric bidirectional) models, the chirality (five times larger emission rate into the right direction in the waveguide as compared to the left direction) can raise the maximum tripartite entanglement value by 35% of by a factor of $\sim 5/14$. Additionally, for both on-resonant and off-resonant cases, chirality aids in maintaining tripartite for a longer duration than the symmetric bidirectional problem. Furthermore, chirality exhibits better robustness against spontaneous emission losses than non-chiral scenarios. Overall, as compared to the single-photon wQED, we conclude that it is the combination of multiphoton Gaussian wavepackets and chiral light-matter interaction that causes the bipartite and tripartite entanglement to take values larger than those found in the single photon wQED.

The rest of the paper is structured as follows. In the next section, we discuss the theoretical description of our system. Next, we introduce the entanglement measure and discuss our results. Finally, we close with a summary section where we also point out possible future directions of this work.

## Theoretical description

### Model

**Figure 1:**
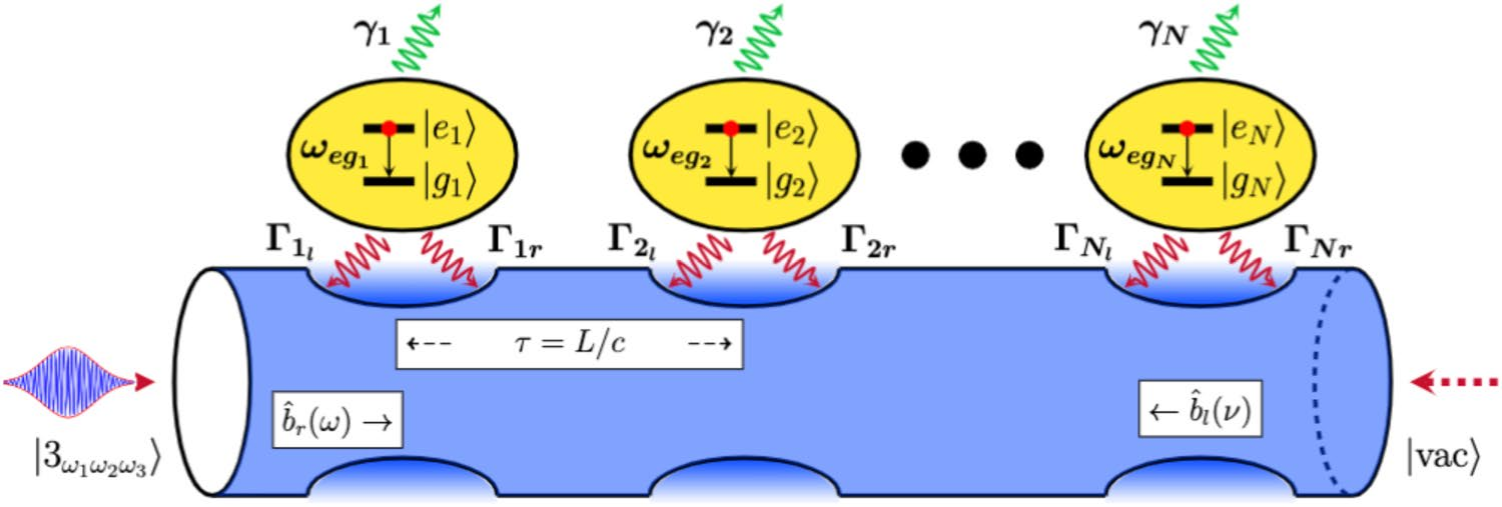
The system studied in this work: A chain of two-level quantum emitters side coupled to a one-dimensional waveguide which is driven by a three-photon wavepacket (represented by the state $|3_{\omega _1\omega _2\omega _3}\rangle$) from the left end of the waveguide. Any photon source does not drive the right end of the waveguide and is, therefore, labeled with a vacuum state $|vac\rangle$. The valleys on the waveguide surface are drawn to indicate the tapered region of the nanofiber where the QEs are trapped to accomplish chiral light-matter interactions. $\gamma _j$ represents the non-waveguide (or spontaneous) emission rate for the *j*th QE. For further details about the system parameters, see the text below.

As shown in Fig. 1, our system consists of a chain of two-level QEs (qubits, quantum dots, artificial atoms, natural atoms, etc.) side coupled to a bidirectional dispersionless and lossless waveguide (tapered fiber). The free Hamiltonian of the emitter chain is given by

1 $$\begin{aligned} \hat{{\mathscr {H}}}_{QE}=\hbar \sum \limits ^{N}_{j=1}{\widetilde{\Delta }}_j\hat{\sigma }^\dagger _j\hat{\sigma }_j, \end{aligned}$$

where ${\widetilde{\Delta }}_j=\omega _{eg_{j}}-\omega _p-i\gamma _j$ is the detuning between the transition frequency $\omega _{eg_{j}}$ of the *j*th QE and the peak frequency $\omega _{p}$ of the three-photon wavepacket. The parameter $\gamma _j$ has been added by hand to account for the spontaneous emission loss from the *j*th QE. Note that in our model, no direct coupling (such as dipole-dipole interaction) is present among the QEs; rather, the interaction is mediated through the waveguide field. $\hat{\sigma }_j\equiv |g_j\rangle \langle e_j|$ is the standard lowering operator for the *j*th QE with $|g_j\rangle (|e_j\rangle )$ being the ground (excited) state. The QE raising and lowering operators follow the standard Ferminonic commutation relation: $\left\{ \hat{\sigma }_i,\hat{\sigma }^\dagger _j \right\} =\delta _{ij}$. Next, we model the waveguide as a collection of two independent multimode quantum harmonic oscillators, one for the left (*l*) direction and the other for the right (*r*) direction. The corresponding photon annihilation operators are labelled as ${\hat{b}}_l(\nu )$ and ${\hat{b}}_r(\omega )$ for the $\nu$th and $\omega$th mode. These operators follow the typical Bosonic commutation relations: $[{\hat{b}}_r(\omega ),{\hat{b}}^\dagger _r(\omega ^{'})]=\delta (\omega -\omega ^{'})$ and $[{\hat{b}}_l(\nu ),{\hat{b}}^\dagger _l(\nu ^{'})]=\delta (\nu -\nu ^{'})$. Thus, the waveguide Hamiltonian $\hat{{\mathscr {H}}}_{w}$ takes the form

2 $$\begin{aligned} \hat{{\mathscr {H}}}_{w}=\hbar \int \limits ^{+\infty }_{-\infty }\omega {\hat{b}}^\dagger _r(\omega ){\hat{b}}_r(\omega )d\omega + \hbar \int \limits ^{+\infty }_{-\infty }\nu {\hat{b}}^\dagger _l(\nu ){\hat{b}}_l(\nu )d\nu . \end{aligned}$$

In $\hat{{\mathscr {H}}}_{w}$, we have considered an infinitely large number of closely spaced waveguide modes such that the integration over all modes is justified. Finally, the following Hamiltonian describes the interaction between the QEs and waveguide field under the rotating wave approximation.

3 $$\begin{aligned} \hat{{\mathscr {H}}}_{int} =&-i\hbar \sum \limits ^N_{j=1}\Big [\int \limits ^{+\infty }_{-\infty } \sqrt{\frac{\Gamma _{jr}}{2\pi }}e^{ik_0d_j}\hat{\sigma }^\dagger _j{\hat{b}}_r(\omega )d\omega + \int \limits ^{+\infty }_{-\infty } \sqrt{\frac{\Gamma _{jl}}{2\pi }}e^{-ik_0d_j}\hat{\sigma }^\dagger _j{\hat{b}}_l(\nu ) d\nu \Big ]+h.c., \end{aligned}$$

where we have assumed $\Gamma _{jr}(\omega )\approx \Gamma _{jr}(\omega _{eg_{j}})\equiv \Gamma _{jr}$ and $\Gamma _{jl}(\omega )\approx \Gamma _{jl}(\omega _{eg_{j}})\equiv \Gamma _{jl}$. Note that in this assumption, we have not applied the Markov approximation (flat bath spectrum around the system resonance)^31,38^; instead, we are considering a highly localized interaction. $d_j$ represents the location of the *j*th emitter with $d_{j+1}-d_{j}=L$ being the separation between two consecutive QEs (or lattice constant) that correspond to the time delay $\tau =L/c$. The parameter $k_0=\omega _{eg}/c$ is the wavenumber associated with the atomic transition frequency, while *c* represents the group velocity of photons in the waveguide. The net Hamiltonian of the global system (QEs, waveguide, and their interaction) is given by $\hat{{\mathscr {H}}}=\hat{{\mathscr {H}}}_{QE} + \hat{{\mathscr {H}}}_{w} + \hat{{\mathscr {H}}}_{int}$.

It is worthwhile to point out that, in this work, we always work in a unit system where detuning is defined in terms of atom-waveguide coupling rate (as we’ll see in the “Results” section) instead of directly linking this coupling rate with the bare atomic frequency. Therefore, it is not possible to directly see the validity of rotating wave approximation under the condition $\Gamma >\omega _{{eg},i}$. We still apply the rotating wave approximation based on the underlying assumption that, typically, the bandwidth of the system resonances is much smaller than the spacing between any two consecutive resonances, allowing us to disregard all the non-resonant terms in the Interaction Hamiltonian. Please note that others have also made such assumptions in the context of deriving Fock state master equations for single and two-photon problems (see, for instance, Ref.^31^ where between Eqs. (1) and (2) of their paper Gheri et al. has some discussion about this point).

### Driven dissipative dynamics

As shown in Fig. 1, the left end of our wQED setup is driven by a reservoir that initially exists in a three-photon Fock state, unlike the standard studied scenario where a classical coherent light source drives the system. Considering this critical distinction, we derive the master equation apt for the present problem and study the driven dissipative dynamics of our wQED setup through the following bi-directional three-photon Fock state master equation.

4a $$ \frac{{d\hat{\rho }_{{3,3}} (t)}}{{dt}} = {\hat{\mathscr{L}}}\left[ {\hat{\rho }_{{3,3}} } \right] + \sum\limits_{{i = 1}}^{N} {\sqrt {\Gamma _{{ir}} } } \left( {\sqrt 3 e^{{ik_{0} d_{i} }} g(t)\left[ {\hat{\rho }_{{2,3}} ,\hat{\sigma }_{i}^{\dag } } \right] + \sqrt 3 e^{{ - ik_{0} d_{i} }} g^{*} (t)\left[ {\hat{\sigma }_{i} ,\hat{\rho }_{{2,3}}^{\dag } } \right]} \right), $$

4b $$ \frac{{d\hat{\rho }_{{2,3}} (t)}}{{dt}} = {\hat{\mathscr{L}}}\left[ {\hat{\rho }_{{2,3}} } \right] + \sum\limits_{{i = 1}}^{N} {\sqrt {\Gamma _{{ir}} } } \left( {\sqrt 2 e^{{ik_{0} d_{i} }} g(t)\left[ {\hat{\rho }_{{1,3}} ,\hat{\sigma }_{i}^{\dag } } \right] + \sqrt 3 e^{{ - ik_{0} d_{i} }} g^{*} (t)\left[ {\hat{\sigma }_{i} ,\hat{\rho }_{{2,2}} } \right]} \right), $$

4c $$ \frac{{d\hat{\rho }_{{1,3}} (t)}}{{dt}} = {\hat{\mathscr{L}}}\left[ {\hat{\rho }_{{1,3}} } \right] + \sum\limits_{{i = 1}}^{N} {\sqrt {\Gamma _{{ir}} } } \left( {e^{{ik_{0} d_{i} }} g(t)\left[ {\hat{\rho }_{{0,3}} ,\hat{\sigma }_{i}^{\dag } } \right] + \sqrt 3 e^{{ - ik_{0} d_{i} }} g^{*} (t)\left[ {\hat{\sigma }_{i} ,\hat{\rho }_{{1,2}}^{\dag } } \right]} \right), $$

4d $$ \frac{{d\hat{\rho }_{{0,3}} (t)}}{{dt}} = {\hat{\mathscr{L}}}\left[ {\hat{\rho }_{{0,3}} } \right] + \sum\limits_{{i = 1}}^{N} {\sqrt {3\Gamma _{{ir}} } } e^{{ - ik_{0} d_{i} }} g^{*} (t)\left[ {\hat{\sigma }_{i} ,\hat{\rho }_{{0,2}}^{\dag } } \right], $$

4e $$ \frac{{d\hat{\rho }_{{2,2}} (t)}}{{dt}} = {\hat{\mathscr{L}}}\left[ {\hat{\rho }_{{2,2}} } \right] + \sum\limits_{{i = 1}}^{N} {\sqrt {\Gamma _{{ir}} } } \left( {\sqrt 2 e^{{ik_{0} d_{i} }} g(t)\left[ {\hat{\rho }_{{1,2}} ,\hat{\sigma }_{i}^{\dag } } \right] + \sqrt 2 e^{{ - ik_{0} d_{i} }} g^{*} (t)\left[ {\hat{\sigma }_{i} ,\hat{\rho }_{{1,2}}^{\dag } } \right]} \right), $$

4f $$ \frac{{d\hat{\rho }_{{1,2}} (t)}}{{dt}} = {\hat{\mathscr{L}}}\left[ {\hat{\rho }_{{1,2}} } \right] + \sum\limits_{{i = 1}}^{N} {\sqrt {\Gamma _{{ir}} } } \left( {e^{{ik_{0} d_{i} }} g(t)\left[ {\hat{\rho }_{{0,2}} ,\hat{\sigma }_{i}^{\dag } } \right] + \sqrt 2 e^{{ - ik_{0} d_{i} }} g^{*} (t)\left[ {\hat{\sigma }_{i} ,\hat{\rho }_{{1,1}} } \right]} \right), $$

4g $$\begin{aligned}{}&\frac{d\hat{\rho }_{0,2}(t)}{dt}=\hat{{\mathscr {L}}}\left[ \hat{\rho }_{0,2}\right] +\sum ^N_{i=1}\sqrt{2\Gamma _{ir}}e^{-ik_0d_i}g^*(t)\left[ \hat{\sigma }_i,\hat{\rho }_{0,1}\right] ,\end{aligned}$$

4h $$\begin{aligned}{}&\frac{d\hat{\rho }_{1,1}(t)}{dt}=\hat{{\mathscr {L}}}\left[ \hat{\rho }_{1,1}\right] +\sum ^N_{i=1}\sqrt{\Gamma _{ir}}\left( e^{ik_0d_i}g(t)\left[ \hat{\rho }_{0,1},\hat{\sigma }^\dagger _i\right] +e^{-ik_0d_i}g^*(t)\left[ \hat{\sigma }_i,\hat{\rho }^\dagger _{0,1}\right] \right) ,\end{aligned}$$

4i $$ \frac{{d\hat{\rho }_{{0,1}} (t)}}{{dt}} = {\hat{\mathscr{L}}}\left[ {\hat{\rho }_{{0,1}} } \right] + \sum\limits_{{i = 1}}^{N} {\sqrt {\Gamma _{{ir}} } } e^{{ - ik_{0} d_{i} }} g^{*} (t)\left[ {\hat{\sigma }_{i} ,\hat{\rho }_{{0,0}} } \right], $$

4j $$\frac{d\hat{\rho }_{0,0}(t)}{dt}=\hat{{\mathscr {L}}}\left[ \hat{\rho }_{0,0}\right].$$

Here, we would like to point out that a similar Fock-state master equation valid for *N* photons has also been reported in the past (see, for example, Eq. (21) in Ref.^32^). However, two main differences exist between our three-photon Fock-state master equation, and the one reported in Ref.^32^. One is the absence of the terms in our master equation that are quadratic in *g*(*t*), which are known to appear in the case of nonlinear interactions (for instance, in cavity quantum optomechanics^39^) or in the case of adiabatically eliminated multi-level quantum systems^40^. Since our problem doesn’t address both scenarios, the absence of such quadratic terms in Eq. (4) is understandable. The second difference stems from the fact that, unlike the master equation reported in Ref.^32^), our master equation incorporates bidirectional couplings between QEs and photon wavepacket, which is suitable for studying wQED problems). The Liouvillian superoperator $\hat{{\mathscr {L}}}$ appearing in the aforementioned equation set (4) and applied to an operator $\hat{\rho }$ consists of three parts

5 $$\begin{aligned} \hat{{\mathscr {L}}}[\hat{\rho }]=\hat{{\mathscr {L}}}_{cs}[\hat{\rho }]+\hat{{\mathscr {L}}}_{pd}[\hat{\rho }]+\hat{{\mathscr {L}}}_{cd}[\hat{\rho }], \end{aligned}$$

with $\hat{{\mathscr {L}}}_{cs}[\hat{\rho }]$, $\hat{{\mathscr {L}}}_{pd}[\hat{\rho }]$, and $\hat{{\mathscr {L}}}_{cd}[\hat{\rho }]$ respectively represent the closed system dynamics, pure decay of energy from the system into the environmental degrees of freedom, and cooperative decay due to collective QE effects. These explicit forms of these Liouvillian subparts are given by

6a $$\begin{aligned}{}&\hat{{\mathscr {L}}}_{cs}[\hat{\rho }]\equiv \frac{-i}{\hbar }\left[ \hat{{\mathscr {H}}}_{QE}, \hat{\rho }\right] ,\end{aligned}$$

6b $$\begin{aligned}{}&\hat{{\mathscr {L}}}_{pd}[\hat{\rho }]\equiv -\sum \limits ^N_{i=1}\Gamma _{irl}\left( \hat{\sigma }^\dagger _i\hat{\sigma }_i\hat{\rho }-2\hat{\sigma }_i\hat{\rho }\hat{\sigma }^\dagger _i+\hat{\rho }\hat{\sigma }^\dagger _i\hat{\sigma }_i\right) ,\end{aligned}$$

6c $$\begin{aligned}{}&\hat{{\mathscr {L}}}_{cd}[\hat{\rho }]\equiv -\sum \limits ^N_{i\ne j=1}\left( \sqrt{\Gamma _{ir}\Gamma _{jr}}~\delta _{i>j}+\sqrt{\Gamma _{il}\Gamma _{jl}}~\delta _{i<j}\right) \left\{ \left(\hat{\sigma }^\dagger _i\hat{\sigma }_j\hat{\rho }-\hat{\sigma }_i\hat{\rho }\hat{\sigma }^\dagger _j\right)e^{-2\pi iD(i-j)}-h.c.\right\} , \end{aligned}$$

where $2\Gamma _{irl}=\Gamma _{ir}+\Gamma _{il}$. The Kronecker delta functions appearing in the expression of $\hat{{\mathscr {L}}}_{cd}[\hat{\rho }]$ are defined as $\delta _{i\gtrless j}=1$, $\forall$ $i\gtrless j$. The parameter *D* represent the ratio of inter-emitter separation *L* and the resonant wavelength $\lambda _0$ where $\lambda _0=2\pi c/\omega _{eg}$. Note that due to the spontaneous emission from the quantum emitters, we did get energy dissipation into the external environment (see Eq. (6b), where part of the Liouvillian results in pure decay (abbreviated with the subscript *pd*)). However, unlike the usual Markov master equations, we obtain time-dependent terms in our Fock state master equation (with prefactor *g*(*t*)). Finally, the explicit form of the various operators appearing in Eq. (4) are given by

7a $$\begin{aligned}{}&\hat{\rho }_{3,3}(t)=\text {tr}_R\left\{ {\hat{U}}(t;t_0)\hat{\rho }_{sys}(t_0)|\Psi _3\rangle \langle \Psi _3|\hat{\rho }_{l}(t_0){\hat{U}}^\dagger (t;t_0)\right\} ,\end{aligned}$$

7b $$\begin{aligned}{}&\hat{\rho }_{j,3}(t)=\text {tr}_R\left\{ {\hat{U}}(t;t_0)\hat{\rho }_{sys}(t_0)|\Psi _j\rangle \langle \Psi _3|\hat{\rho }_{l}(t_0){\hat{U}}^\dagger (t;t_0)\right\} ,\end{aligned}$$

7c $$\begin{aligned}{}&\hat{\rho }_{j,2}(t)=\text {tr}_R\left\{ {\hat{U}}(t;t_0)\hat{\rho }_{sys}(t_0)|\Psi _j\rangle \langle \Psi _2|\hat{\rho }_{l}(t_0){\hat{U}}^\dagger (t;t_0)\right\} ,\end{aligned}$$

7d $$\begin{aligned}{}&\hat{\rho }_{k,1}(t)=\text {tr}_R\left\{ {\hat{U}}(t;t_0)\hat{\rho }_{sys}(t_0)|\Psi _k\rangle \langle \Psi _1|\hat{\rho }_{l}(t_0){\hat{U}}^\dagger (t;t_0)\right\} ,\end{aligned}$$

7e $$\begin{aligned}{}&\hat{\rho }_{0,0}(t)=\text {tr}_R\left\{ {\hat{U}}(t;t_0)\hat{\rho }_{sys}(t_0)|vac\rangle \langle vac|\hat{\rho }_{l}(t_0){\hat{U}}^\dagger (t;t_0)\right\} . \end{aligned}$$

Here $$ j=2,1,0,$$, $$k=1,0$$, $${\hat{U}}(t;t_0) \equiv \exp [\frac{-i}{\hbar }\hat{{\mathscr {H}}}(t-t_0)]$$ is the time evolution operator with $$\hat{{\mathscr {H}}}$$ being the total Hamiltonian defined in the paragrap below Eq. (3), and $$\hat{\rho }_l(t)$$ is the density operator for the left continuum in the waveguide. $$|\Psi _3\rangle $$, $$|\Psi _2\rangle $$ and $$|\Psi _1\rangle $$ are the three-, two- and one-photon reservoir states, respectively with $$|\Psi _0\rangle =|vac\rangle $$. Note that only the diagonal operators can be categorized as physically valid density matrices in the above-mentioned set of operators. The rest of the off-diagonal operators are not density matrices but they do obey a useful property that $$\hat{\rho }^\dagger _{j,3}(t) = \hat{\rho }_{3,j}(t)$$, $$\hat{\rho }^\dagger _{j,2}(t) = \hat{\rho }_{2,j}(t)$$, and $$\hat{\rho }^\dagger _{k,1}(t)=\hat{\rho }_{1,k}(t)$$.

### Initial conditions

Initially, we consider all QEs to be in their ground state with the right waveguide continuum in a three-photon wavepacket with the joint spectral density function ${\mathscr {G}}(\omega _1,\omega _2,\omega _3)$ and the left continuum in a vacuum state i.e. the initial pure state $|\Psi \rangle$ of the system and environment can be expressed as a product state of the form:

8 $$\begin{aligned}{}&|\Psi \rangle =|\Psi _{QE}\rangle \otimes |\Psi _r\rangle \otimes |\Psi _l\rangle = \bigotimes _j|g_j\rangle \otimes |\Psi _r\rangle \otimes |vac\rangle ,\nonumber \\&\textit{with}~ |\Psi _{r}\rangle =\frac{1}{\sqrt{3!}}\int \limits ^{+\infty }_{-\infty }\int \limits ^{+\infty }_{-\infty }\int \limits ^{+\infty }_{-\infty } d\omega _1 d\omega _2 d\omega _3 ~{\mathscr {G}}(\omega _1,\omega _2,\omega _3){\hat{b}}^\dagger _r(\omega _1){\hat{b}}^\dagger _r(\omega _2){\hat{b}}^\dagger _r(\omega _2)|vac\rangle . \end{aligned}$$

At this stage we keep the form of ${\mathscr {G}}(\omega _1,\omega _2,\omega _3)$ general, however, the normalization condition on $|\Psi \rangle$ requires any ${\mathscr {G}}(\omega _1,\omega _2,\omega _3)$ must follow the condition

9 $$\begin{aligned} \int \limits ^{+\infty }_{-\infty }\int \limits ^{+\infty }_{-\infty }\int \limits ^{+\infty }_{-\infty } \left| {\mathscr {G}}(\omega _1,\omega _2,\omega _3)\right| ^2 d\omega _1 d\omega _2 d\omega _3=1, \end{aligned}$$

where in arriving at this condition, we have assumed the spectral function ${\mathscr {G}}(\omega _1,\omega _2,\omega _3)$ is symmetric under the exchange of mode frequencies $\omega _1$, $\omega _2$, and $\omega _3$. Finally, we impose

10 $$\begin{aligned}{}&\hat{\rho }_{m,m}(0)=\hat{\rho }_{sys}(0)=\bigotimes _j|g_j\rangle \langle g_j|, \forall m~\text {and}~n=0,1,2,3;~\hat{\rho }_{m,n}(0)=0,~\text {with}~m\ne n, \end{aligned}$$

which are the initial conditions followed by the operators appearing in Eq. (4).

Before moving on to the “Results and discussion” section, we would like to explain how our Fock state master equation can be regarded as non-Markovian. To this end, we first consider a single-photon Fock state master equation where the initial state of the reservoir can be expressed as:

11 $$\begin{aligned} |\Psi ^{(1)}_r\rangle =\int ^\infty _{-\infty }{\mathscr {G}}(\omega ){\hat{b}}^\dagger _r(\omega )|vac\rangle d\omega . \end{aligned}$$

Following the above notation, the ‘r’ subscript indicates the right direction in the waveguide, with superscript (1) representing the single-photon nature of the state here. ${\mathscr {G}}(\omega )$ is the spectral function for the single photon wavepacket. Now we notice that if we apply the environment input operator ${\hat{b}}_{in}$ (as introduced in Ref.^31^) on this state, we obtain

12 $$\begin{aligned} {\hat{b}}_{in}|\Psi ^{(1)}_r\rangle = \frac{1}{\sqrt{2\pi }}\int ^\infty _{-\infty }e^{-i\omega ^{'}(t-t_0)}{\hat{b}}_{r}(\omega ^{'})d\omega ^{'}|\Psi ^{(1)}_r\rangle = \widetilde{{\mathscr {G}}}(t)|vac\rangle , \end{aligned}$$

while performing the above calculation we have used the commutation relation $[{\hat{b}}_{r}(\omega ),{\hat{b}}^\dagger _{r}(\omega ^{'})]=\delta (\omega -\omega ^{'})$ and finally defined $\widetilde{{\mathscr {G}}}(t)= \sqrt{2\pi }\int ^\infty _{-\infty }{\mathscr {G}}(\omega )e^{-i\omega (t-t_0)}d\omega$. On the other hand, if we have a three-photon wavepacket launched from the environment into the right direction of the waveguide, as reported in Eq. (8), and we now apply the input operator to this state, after some calculation, one can find

13 $$\begin{aligned} {\hat{b}}_{in}|\Psi _r\rangle&= \sqrt{3} \int ^\infty _{-\infty }\int ^\infty _{-\infty } \left( \int ^\infty _{-\infty }\frac{1}{\sqrt{2\pi }}{\mathscr {G}}(\omega _1,\omega _2,\omega ^{'})e^{-i\omega ^{'}(t-t_0)}d\omega ^{'}\right) {\hat{b}}^\dagger _r(\omega _1){\hat{b}}^\dagger _{r}(\omega _2)|vac\rangle d\omega _1 d\omega _2\nonumber \\&\equiv \sqrt{3} \int ^\infty _{-\infty }\int ^\infty _{-\infty }\widetilde{{\mathscr {G}}}(\omega _1,\omega _2,t){\hat{b}}^\dagger _r(\omega _1){\hat{b}}^\dagger _{r}(\omega _2)|vac\rangle d\omega _1 d\omega _2, \end{aligned}$$

while arriving at the final expression, we have assumed Bosonic symmetry under the exchange of $\omega _i \longleftrightarrow \omega _j$, $\forall i=1,2,3$ and $j=1,2,3$ with $i\ne j$. Note that, under the narrow-bandwidth assumption (as explained in Ref.^31^ and Ref.^33^) the function $\widetilde{{\mathscr {G}}}(\omega _1,\omega _2,t)$ leads us to the three-photon Gaussian wavepacket *g*(*t*) which shows up in our final Fock state master equation (see Eq. (4)). Two key differences can be immediately noticed in comparison to the single-photon and vacuum environment state cases. These differences explain why Eq. (4) has non-Markovian features.

1. For the three-photon wavepackets, the application of the input operator produces a state that carries the information left in the field about the destruction of a single photon at a certain frequency encoded in the function $\widetilde{{\mathscr {G}}}(\omega _1,\omega _2,t)$. This behavior is unlike the problem of the single-photon case where ${\hat{b}}_{in}|\Psi ^{(1)}_r\rangle$ was not able to store the information about the destruction of the photon in the field. Note that this behavior extends to purely vacuum states environments as well as ${\hat{b}}_{in}|vac\rangle =0$ doesn’t produce any time-dependent terms in the resultant master equation.
2. In the three-photon problem, the resultant state (which now has two photons) evolves in a non-trivial fashion after applying the input operator. It generates the hierarchy of new operators defined in Eq. (7), again unlike the single-photon or vacuum environment cases where the application of ${\hat{b}}_{in}$ produces either a vacuum state or give zero contribution, which in the case of vacuum state environments produce Markovian results.

## Results and discussion

In this section, by numerically solving our three-photon bidirectional Fock state master equation, we address two questions: (1) How does the incoming three-photon wavepacket excite the QEs, and how does the population evolve in time? (2) How does the photon absorption & emission generate entanglement among QEs, and how can chirality impact the entanglement manipulation? Albeit Eq. (4) is valid for any number of QEs, in the following, we focus on situations up to 3 QEs. To set the stage, we begin with the most straightforward possible problem of a single QE.

### One QE case and population dynamics

**Figure 2:**
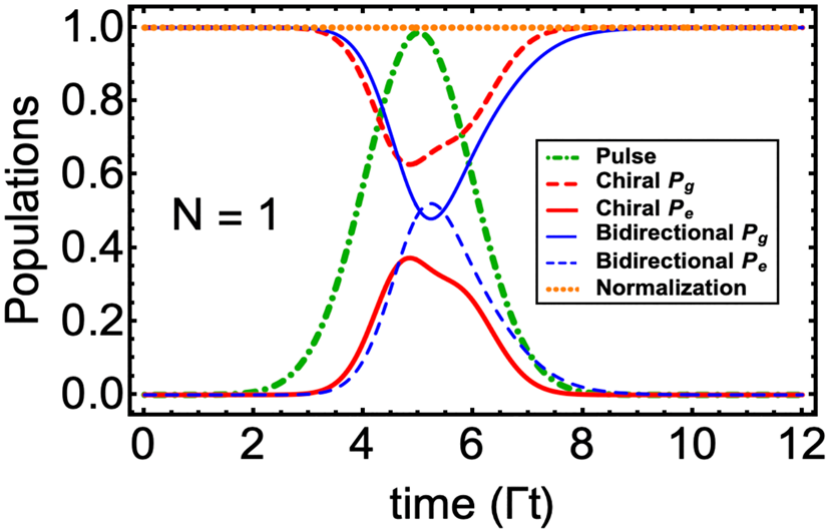
Population dynamics, quantified in the $\Gamma ^{-1}$ units, for a single ($N=1$) two-level QE when interacted with a three-photon Gaussian wavepacket. We have considered the following common parameters in all curves: $\Delta =0$, $\mu =1.46\Gamma$, ${\overline{t}}=5\Gamma ^{-1}$. For the chiral case, we have set $\Gamma _{1r}/\Gamma _{1l}=5$; while for the bidirectional case, we have selected a symmetric case, i.e., $\Gamma _{1r}=\Gamma _{1l}\equiv \Gamma$. The orange dotted horizontal line confirms normalization in both bidirectional and chiral cases.

For the single-QE case ($\mathrm{N=1}$) our free QE Hamiltonian reduces to ${\hat{\mathscr {H}}}_{QE}=\hbar {\widetilde{\Delta }}\hat{\sigma }^\dagger \hat{\sigma }$ and as initial conditions we assume $\hat{\rho }_{m,m}(t_0)=|g\rangle \langle g|$, $\forall m=3,2,1,0$ and the remaining operators to be zero. For the three-photon spectral density function ${\mathscr {G}}(\omega _1,\omega _2,\omega _3)$, we assume a factorized form such that using Schmidt decomposition, one can write

14 $$\begin{aligned} {\mathscr {G}}(\omega _1,\omega _2,\omega _3)=\frac{1}{\sqrt{3!}}\sum _{\textrm{cyc}}g_1(\omega _1)g_2(\omega _2)g_3(\omega _2), \end{aligned}$$

with $\sum _{\textrm{cyc}}$ representing the sum over all pairwise cyclic permutations of the indices, which counts to 6 terms. We point out that the type mentioned above of decomposition of the spectral density function is experimentally achievable when the three-photon wavepacket is generated by combining the single photons emitted by three independent sources^31,41^. Moving forward, in all plots to follow, we select a real-valued Gaussian temporal profile for each *g* function, i.e.

15 $$\begin{aligned} g(t)=\frac{\sqrt{\mu }}{(2\pi )^{1/4}}\exp \left( -\frac{\mu ^2}{4}\left( t-{\overline{t}}~\right) ^2\right) . \end{aligned}$$

Here $\mu$ and ${\overline{t}}$ represent the standard deviation and mean of the Gaussian function, respectively. In Fig. 2 we plot the population dynamics under strong drive condition i.e. $|\Omega ^{(max)}(t)|>\Gamma$ with $\Omega (t)=\sqrt{2\Gamma }g(t)$. The rest of the parameters mentioned in the plot caption are selected to generate higher excitation probabilities^32,42^. The green dotted dashed curve shows our three-photon normalized Gaussian wavepacket peaked at $t={\overline{t}}=5\Gamma ^{-1}$. We have plotted the ground ($P_g$) and excited population ($P_e$) for two cases, namely, a non-chiral or symmetric bidirectional coupling ($\Gamma _{1r}=\Gamma _{1l}$) case (thin blue solid and dashed curves); and a chiral case (thick red solid and dashed curves) in which emission in the right direction is five-time larger than the left direction ($\Gamma _{1r}=5\Gamma _{1l}$). In both cases, we note that as the Gaussian wavepacket begins interacting with the QE, it took almost $t\sim \Gamma ^{-1}$ time before the populations change.

**Figure 3:**
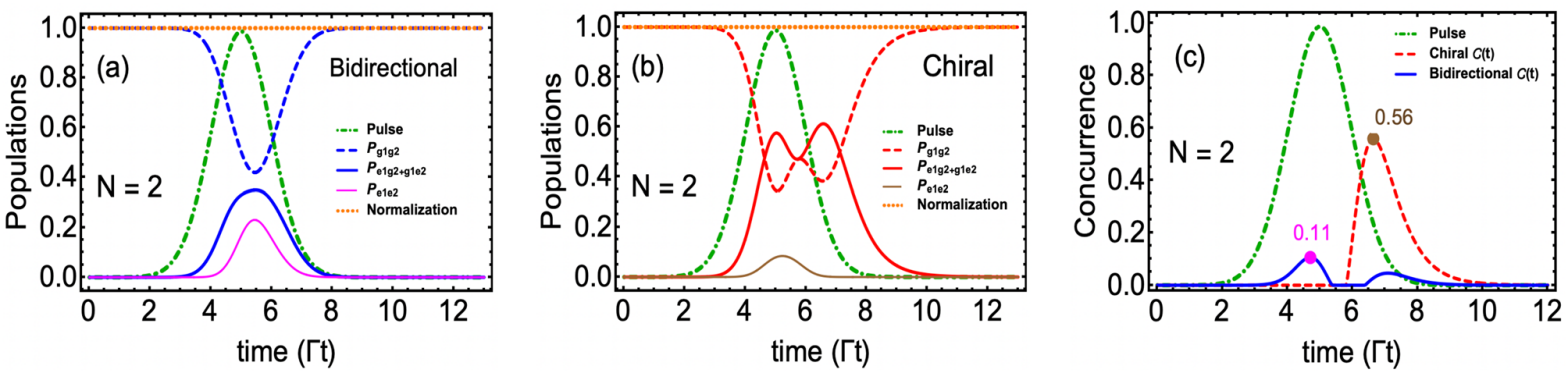
Two-emitter $\mathrm{N=2}$ wQED driven by a three-photon Gaussian wavepacket. Population dynamics in (**a**) Bidirectional case $\Gamma _{ir}=\Gamma _{il}=1$ and (**b**) Chiral case $\Gamma _{ir}/\Gamma _{il}=5$, $\forall i=1,2$. In the subscripts of *P*, the first and second slots identify the state of the first and second QE, respectively. (**c**) Entanglement/concurrence evolution in both bidirectional and chiral cases. Pink and brown-colored dots have identified the location and the maximum value of entanglement. For the sake of simplicity, all QEs are assumed to be identical, and time delays have been ignored. The rest of the parameters are the same as in Fig. 2.

The maximum value of the excited state probability $P^{(max)}_e$ attained for the bidirectional case turns out to be 0.521 at $t=5.25\Gamma ^{-1}$, which is smaller than the reported value of 0.801^32^ for the single photon problem due to the involvement of bidirectional decays in our model. Additionally, the shape of $P_e$ follows the profile of Gaussian input, which decays as the photon wavepacket leaves the QE region. Conversely, the chiral case allowed to attain a smaller value of $P^{(max)}_e=0.373$ due to a higher decay rate into the right waveguide direction. This maximum value is achieved at a time $t=4.85\Gamma ^{-1}$ slightly before the $P_e$ reaches its maximum value for the bidirectional case. More importantly, we observe the formation of a side shoulder around $t\sim 5.5\Gamma ^{-1}$. Such behavior of $P_e$ in the chiral case is known for the single and two-photon wQED problems^22,23^ and (as discussed below) will help in better emitter-emitter entanglement generation and control.

### Two-QE case and bipartite entanglement

We now extend our wQED study to two QEs. In addition to new ways of population distribution, the case of two QEs opens the possibility of generating entanglement between the QEs, which we quantify through the well-known concurrence measure^43,44^. For two particles, say particle *A* and particle *B*, existing in a bipartite pure or mixed state $\hat{\rho }_{AB}$, Wootter’s concurrence ${\mathscr {C}}_{A(B)}$ is defined as

16 $$\begin{aligned} {\mathscr {C}}_{A(B)} = \max \left( 0,\sqrt{\lambda _1}-\sqrt{\lambda _2}-\sqrt{\lambda _3}-\sqrt{\lambda _4}\right) , \end{aligned}$$

where eigenvalues of operator ${\widetilde{\rho }}_{AB}$, $\lambda _i$, $\forall i=1,2,3,4$ are written in a descending order. ${\widetilde{\rho }}_{AB}$ is called the spin-flipped density operator, which is related to the system density operator and the Pauli spin-flip operator $\hat{\sigma }_y$ through

17 $$\begin{aligned} {\widetilde{\rho }}_{AB}=\hat{\rho }_{AB}\left( \hat{\sigma }_y\otimes \hat{\sigma }_y\right) \hat{\rho }^*_{AB}\left( \hat{\sigma }_y\otimes \hat{\sigma }_y\right) . \end{aligned}$$

The concurrence bounds are defined as $0\le {\mathscr {C}}_{A(B)}\le 1$ with ${\mathscr {C}}_{A(B)}=1$ refers to a maximally entangled bipartite state (for example, a Bell state^45^) and ${\mathscr {C}}_{A(B)}=0$ indicates an entirely separable (unentangled) state. For the present problem we introduce the basis set $\lbrace |g_1g_2\rangle \rightarrow |1\rangle , |e_1g_2\rangle \rightarrow |2\rangle , |g_1e_2\rangle \rightarrow |3\rangle , |e_1e_2\rangle \rightarrow |4\rangle \rbrace$. Next, subject to the initial condition $\hat{\rho }_{sys}(0)=|g_1g_2\rangle \langle g_1g_2|$, we numerically solve the three-photon Fock state master equation. Therein, we find that the spin-flip density matrix of the two-QE system takes the following form, with 8 out of 16 time-dependent density matrix elements remaining zero for all times

18 $$\begin{aligned} {\widetilde{\rho }}_{12}(t)= \begin{pmatrix} \rho _1\rho _{16}+\rho ^2_4 &{} 0 &{} 0 &{} \rho _1\rho _4\\ 0 &{} 2\rho ^2_6 &{} 2\rho ^2_6 &{} 0\\ 0 &{} 2\rho ^2_6 &{} 2\rho ^2_6 &{} 0\\ \rho _1\rho _4 &{} 0 &{} 0 &{} \rho _{1}\rho _{16} \end{pmatrix}. \end{aligned}$$

Note that we have adopted short notation here in which $\rho _1\equiv \langle 1|\hat{\rho }_{3,3}(t)|1\rangle$, $\rho _4\equiv \langle 1|\hat{\rho }_{3,3}(t)|4\rangle$, $\rho _6\equiv \langle 2|\hat{\rho }_{3,3}(t)|2\rangle$, and $\rho _{16}\equiv \langle 4|\hat{\rho }_{3,3}(t)|4\rangle$. Diagonalization of ${\widetilde{\rho }}_{12}(t)$ yields the following set of eigenvalues

19 $$\begin{aligned}{}&\lambda _1 = 0, ~~\lambda _2 = 4\rho ^2_6, \lambda _3 = \rho _1\rho _{16}+\frac{1}{2}\rho _4\left( \rho _4-\sqrt{\rho ^2_4+4\rho _1\rho _{16}}\right) ,~\text {and}~\lambda _4 = \rho _1\rho _{16}+\frac{1}{2}\rho _4\left( \rho _4+\sqrt{\rho ^2_4+4\rho _1\rho _{16}}\right) . \end{aligned}$$

Inserting these eigenvalues in Eq. (16), one can find the entanglement between QEs. In Fig. 3c, we plot this bipartite entanglement in both the bidirectional symmetric and chiral cases. In parts (a) and (b) of Fig. 3, the populations corresponding to these two cases have also been plotted. For the bidirectional symmetric case, we notice that the temporal profile of concurrence follows a pattern with two peaks (at $t=4.70\Gamma ^{-1}$ and $t=6.65\Gamma ^{-1}$) separated by a dip (centered at $t\sim 6\Gamma ^{-1}$) while reaching the maximum value of up to $11\%$. The first peak is reached just before the three-photon wavepacket reaches its maximum value. After that, as the wavepacket begins to leave the emitter region, we observe an increase in the concurrence around $t= 6.65\Gamma ^{-1}$, forming the second peak. To fully understand the behavior of this pattern, not only the population dynamics needs to be discussed (in terms of the formation of two types of Bell states, namely, $\left( |g_1g_2\rangle +|e_1e_2\rangle \right) /\sqrt{2}$ and $\left( |e_1g_2\rangle +|g_1e_2\rangle \right) /\sqrt{2}$) but also the coherence terms’ time evolution needs to be analyzed. We plan to include the discussion of coherence terms in the future continuation of this work.

Next, in the chiral case ($\Gamma _{ir}=5\Gamma _{il}$, i=1,2), we find a marked change in the behavior of population and entanglement dynamics compared to the bidirectional symmetric case. On the one hand, in Fig. 3b, we observe $P_{e_1g_2+g_1e_2}$ (red solid curve) exhibiting a two-peak pattern with a maximum value increase by a factor of almost two compared to the symmetric coupling case (blue solid thick curve in Fig. 3a). On the other hand, the maximum value of both QEs excited probability $P_{e_1e_2}$ (brown solid thin curve in Fig. 3b) reduced more than 1/2 compared to the symmetric problem. We find that this single QE excited probability trend extended down to the entanglement behavior as well, where the concurrence in the chiral case (dashed red curve in Fig. 3c) showing a single peak pattern but with five times higher value achieved for the maximum entanglement. Furthermore, we note that chirality also assisted in sustaining this entanglement for times between $8\Gamma ^{-1}$ to $10\Gamma ^{-1}$ even after the three-photon wavepacket diminishes.

### Three-QE case and tripartite entanglement

**Figure 4:**
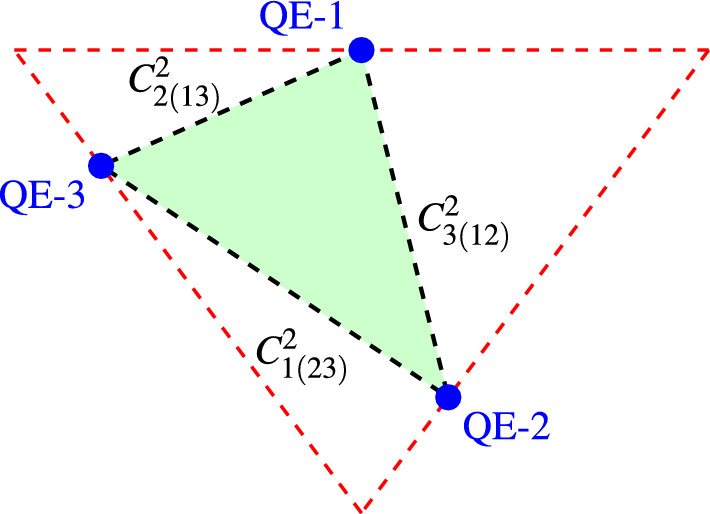
The concurrence triangle for a tripartite system (composed of QE-1, 2, and 3). Note that the length of each side of the triangle is equal to the square of the concurrence between different possible bipartite pairings.

**Figure 5:**
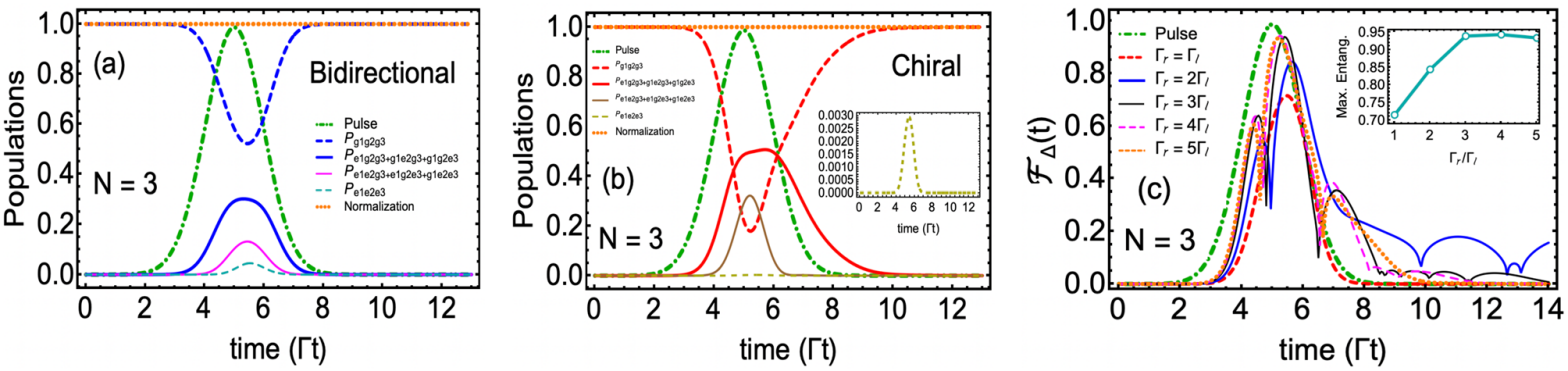
Population dynamics for the three-photon three-QE ($N=3$) problem. (**a**) Symmetric bidirectional case i.e. $\Gamma _{ir}=\Gamma _{il}=1$; and (**b**) Chiral case with $\Gamma _{ir}/\Gamma _{il}=5$, $\forall i=1,2,3$. Similar to Fig. 3, all QEs are assumed to be identical, and time delays have been ignored. The plot legends follow the notation in which the first, second, and third slots in the subscripts represent the state of the first, second, and third QE, respectively. The inset in the plot (**b**) represents the curve of all three QEs being excited simultaneously ($P_{e_1e_2e_3}$). (**c**) Time evolution of tripartite entanglement among three QEs quantified through the concurrence fills ${\mathscr {F}}_\Delta (t)$ measure. Emitter-waveguide coupling strength in the right direction $\Gamma _r$ has been varied in units of $\Gamma _l$. The inset shows the behavior of maximum entanglement (${\mathscr {F}}_{\Delta , max}$) achieved for each chosen value of $\Gamma _r/\Gamma _l$. The rest of the parameters in all plots are the same as used in Fig. 2.

**Table 1:** Maximum excitation probability comparison.

**Excitation**	**Bidirectional**			**Chiral**		
	N=1	N=2	N=3	N=1	N=2	N=3
$P_{1,max}$	0.52 at $5.25\Gamma ^{-1}$	0.35 at $5.47\Gamma ^{-1}$	0.30 at $5.31\Gamma ^{-1}$	0.37 at $4.85\Gamma ^{-1}$	0.61 at $6.57\Gamma ^{-1}$	0.51 at $5.71\Gamma ^{-1}$
$P_{2,max}$		0.23 at $5.44\Gamma ^{-1}$	0.13 at $5.44\Gamma ^{-1}$		0.08 at $5.22\Gamma ^{-1}$	0.32 at $5.20\Gamma ^{-1}$
$P_{3,max}$			0.05 at $5.50\Gamma ^{-1}$			0.003 at $5.41\Gamma ^{-1}$

Moving on to the three-QE mixed states, it turns out that the bipartite concurrence measure doesn’t extend down straightforwardly to the tripartite case^46,47^. To this end, we apply a recently proposed tripartite entanglement measure by Xie and Eberly^34^. This measure is reported to quantify genuine three-party entanglement by analyzing the area of the concurrence triangle (hence the name triangle measure or concurrence fill). The measure itself involves calculating the pairwise concurrence among all three QEs with a bipartite-split between *i*th qubit (treated as one subsystem) and *j*, *k* qubit pair (as the other subsystem) as shown in Fig. 4). For the set of qubits *i*, *j*, *k*; such a “one-to-other” concurrence is known to follow the identity^48^

20 $$\begin{aligned} {\mathscr {C}}^2_{i(jk)} \le {\mathscr {C}}^2_{j(ki)} + {\mathscr {C}}^2_{k(ij)}, \end{aligned}$$

where, for example, ${\mathscr {C}}^2_{1(23)}$ is calculated using^49^.

21 $$\begin{aligned} {\mathscr {C}}^2_{1(23)}=\sqrt{2(1-\text {tr}\lbrace \hat{\rho }^2_1\rbrace )}~~ \text {with}~~ \hat{\rho }_1:=\text {tr}_{23}\lbrace \hat{\rho }_{123} \rbrace . \end{aligned}$$

$\hat{\rho }_1$ in the last equation represents the reduced density matrix of the first qubit obtained by tracing out the second and third qubit from the total system density matrix $\hat{\rho }_{123}$. Thus, considering ${\mathscr {C}}^2_{1(23)}$, ${\mathscr {C}}^2_{2(31)}$, and ${\mathscr {C}}^2_{3(12)}$ as lengths of the side of a triangle, Xie and Eberly used Heron’s expression for the area of such a triangle and arrived at the following formula that describes the triangle measure:

22 $$\begin{aligned}{}&{\mathscr {F}}_\Delta = \left[ \frac{16}{3} {\mathscr {Q}}\left( {\mathscr {Q}}-{\mathscr {C}}^2_{1(23)}\right) \left( {\mathscr {Q}}-{\mathscr {C}}^2_{2(13)}\right) \left( {\mathscr {Q}}-{\mathscr {C}}^2_{3(12)}\right) \right] ^{\frac{1}{4}},~~\text {where}~~~{\mathscr {Q}}=\frac{1}{2}\left( {\mathscr {C}}^2_{1(23)}+{\mathscr {C}}^2_{2(13)}+{\mathscr {C}}^2_{3(12)}\right) , \end{aligned}$$

where the prefactor $\left( 16/3\right) ^{1/4}$ ensures that ${\mathscr {F}}_\Delta$ remains bounded between 0 and 1, again 1 referring to the maximum of genuinely entangled tripartite state (such as W or GHZ state^30^) and 0 indicates a fully unentangled state. Furthermore, consistent with Fig. 4, ${\mathscr {Q}}$ is also called the half-perimeter of the concurrence triangle.

In Fig. 5, we plot population and entanglement dynamics for the three-QEs problem. In Fig. 5a and b, we compare the populations in bidirectional symmetric and chiral scenarios, respectively. With the presence of the third QE, all probabilities including single emitter being excited ($P_{e_1g_2g_3+g_1e_2g_3+g_1g_2e_3}$), double emitter excited ($P_{e_1e_2g_3+e_1g_2e_3+g_1e_2e_3}$) and triple emitter excited ($P_{e_1e_2e_3}$) have been reported. In both bidirectional and chiral scenarios, we note that the corresponding probability shows a considerable reduction as the number of excited QEs increases. In particular, in the chiral case, $P_{e_1e_2e_3}$ becomes too tiny such that we have to include it as the inset in Fig. 5b where it reaches a maximum value of merely $0.3\%$. As summarized in Table 1, we find that the maximum value probability of one- ($P_{1,max}$) and two- ($P_{2,max}$) QE excited in the bidirectional model shows a noticeable decrease for $N=3$ case as compared to the respective $N=1$ and $N=2$ cases. However, in the chiral case, such a trend is broken. Additionally, by the comparison of Figs. 3b and 4b, we notice that unlike $N=2$ problem with chiral couplings, $N=3$ chiral scenario fails to show any oscillatory behavior in the populations. But single excitation probability $P_{e_1g_2g_3+g_1e_2g_3+g_1g_2e_3}$ forms an almost plateau between $5\Gamma ^{-1}\lesssim t \lesssim 6.5\Gamma ^{-1}$ which helps $P_{e_1g_2g_3+g_1e_2g_3+g_1g_2e_3}$ to maintain a non-zero value for an additional $t\cong 1.5\Gamma ^{-1}$ after the complete diminishing of the three-photon pulse.

**Figure 6:**
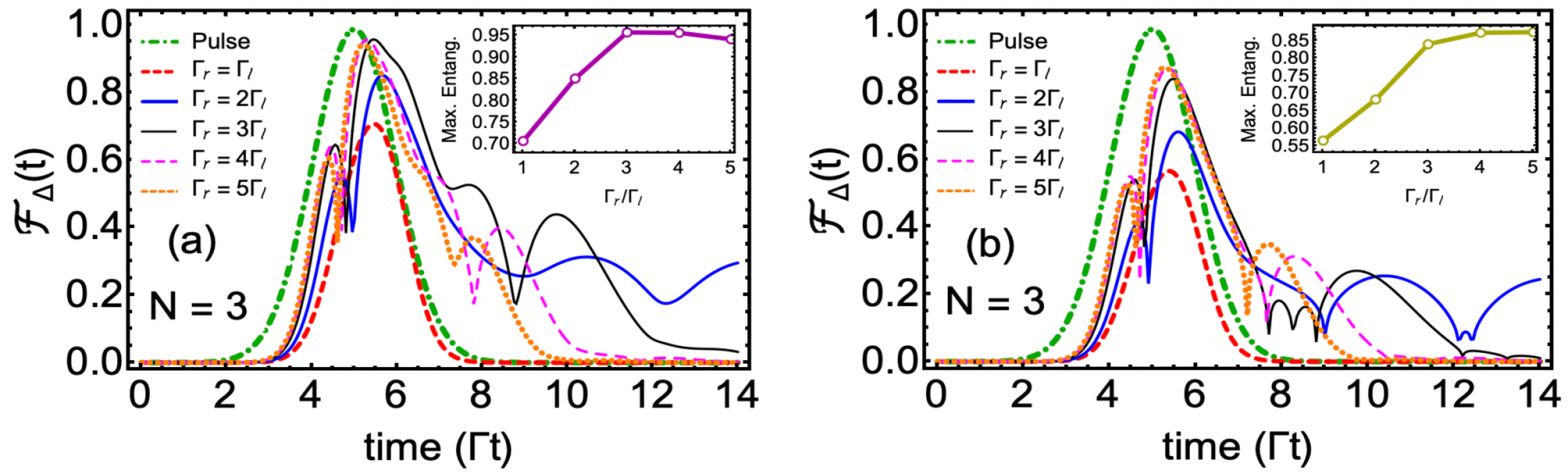
(**a**) Time evolution of tripartite entanglement when all QEs’ transition frequency is detuned by $\Gamma /2$ from the peak frequency of the three-photon wavepacket. Here, we have set $\Gamma _l\equiv \Gamma$. (**b**) Entanglement dynamics in the presence of spontaneous emission rate $\gamma$, which is assumed to be the same for all QEs with a value of $3\Gamma /4$. Insets in both plots show the maximum entanglement as a function of $\Gamma _r$. Besides detuning and spontaneous emission rate, all parameters are the same as previously.

In Fig. 5c, we plot the time evolution of concurrence fill while varying the right direction emitter-waveguide coupling $\Gamma _r$ (assumed to be the same for all QEs) from symmetric bidirectional case $\Gamma _r=\Gamma _l$ to the maximum chiral case $\Gamma _r=5\Gamma _l$. We notice, following the population trend observed in Fig. 5a and b, for all non-chiral cases, the entanglement among QEs survives for a time longer than the pulse duration. Additionally, the irregular oscillations in ${\mathscr {F}}_\Delta (t)$ for chiral case exhibit the phenomenon of entanglement collapse and revival^50–52^ which is more visible for the $\Gamma _r=3\Gamma _l$ case (thin blue curve). Most importantly, we notice that the maximum value achieved by the entanglement in all chiral cases poses an upper bound on the maximum value of entanglement achieved in the symmetric directional case where ${\mathscr {F}}_\Delta \cong 0.70$. This important finding is further emphasized in the inset plot in Fig. 5c, where we observe this maximum value to be elevated by more than 35% as we go from the symmetric bidirectional case of $\Gamma _r$ to chiral cases of $3\Gamma _l\le \Gamma _r\le 5\Gamma _l$. Note that for single-photon two-qubit wQED problem, Ballestero et al. have shown that the chirality can be used to enhance the maximum entanglement by a factor of 3/2 as compared to the corresponding symmetric bidirectional case^19^. Similarly, Mirza et al. (the corresponding author of this work) have reported the twice enhancement in qubit-qubit entanglement for the two-photon two-qubit case^23^. We, on the other, in this work, have shown that this trend extends down to genuine tripartite entanglement where $\Gamma _r\ge 3\Gamma _l$ case chirality assists in increasing the concurrence fill among three-QEs by 35% (factor of $\sim 5/14$).

The appearance of entanglement collapse and revival pattern, as observed in Fig. 5c, can be attributed to the non-Markovian nature of our Fock state master equation. However, in contrast to the typically studied non-Markovian master equations^53^ (for instance, the time-convolutionless type of master equations, which are local in time, or the Nakajima-Zwanzig master equation, which has an integro-differential form), in our case, the time dependence in certain terms of our Fock state master equations emerges due to the input operator’s application on the environment’s three-photon state. Additionally, since our three-photon wavepacket drives the system strongly, i.e., $|\sqrt{2\Gamma }g(t)|_{max} > \Gamma$ with $\Gamma _r=\Gamma _l=\Gamma$, that can also lead to the non-Markovianity induced collapse and revival pattern of entanglement in our study.

Finally, we point out that the reason for achieving higher entanglement in the tripartite case compared to the bipartite case relies on the entanglement measure we use. In particular, in quantifying the tripartite entanglement, the definition of so-called “one-to-other” concurrence (see Eq. (21)) and hence the triangle measure (see Eq. (22)) works only for the pure states. However, our qubit system (after eliminating the waveguide field) exists in a mixed state. From this point of view, the reported entanglement values for the tripartite case (Figs. 5c and 6 are essentially the best possible values any mixed state can attain as soon as the mixed state reaches its pure state counterpart. On the other hand, for the bipartite case, we use Wootter’s definition of concurrence (see Eq. (16)), which is known to work for mixed states. Thus, our reported entanglement is not the best scenario value for the bipartite case.

### Tripartite entanglement in the presence of detuning and spontaneous emission

So far, we have assumed an on-resonance scenario where the peak frequency of the three-photon wavepacket $\omega _p$ has been set equal to the emitter transition frequency $\omega _{eg}$. Additionally, we have completely ignored the photon emissions into non-waveguide modes through spontaneous emission. We now address these two scenarios separately and plot the three-QE entanglement dynamics for a detuned case with no spontaneous emission (i.e., $\omega _p-\omega _{eg}=\Gamma /2$ and $\gamma =0$) in Fig. 6a and for an on-resonance case with a non-zero spontaneous emission scenario ($\omega _p=\omega _{eg}$ and $\gamma = 3\Gamma /4$) in Fig. 6b.

From Fig. 6a, we note that for all cases, as we increase $\Gamma _r$ value from $\Gamma _l$ to $5\Gamma _l$, near the peak frequency of the wavepacket, detuning preserves the overall profile of the entanglement observed in the on-resonance situation. Additionally, the inset plot shows that the maximum entanglement values follow a similar pattern as found in the no-detuning problem. However, we observe the novel aspect of Fig. 6a in a long time ($t\gtrsim 8\Gamma ^{-1}$) behavior of ${\mathscr {F}}_\Delta (t)$ where tripartite entanglement sustains for longer times and tend to produce more oscillatory behavior as compared to the no-detuning problem (compare, for instance, thin black ($\Gamma _r=3\Gamma _l$) curves in Figs. 5c and 6a).

In Fig. 6b, we study the impact of spontaneous emission on the tripartite entanglement under the strong coupling regime of wQED ($\gamma < \Gamma$). As expected, we find that a finite spontaneous emission considerably reduced the entanglement while keeping the overall profile of entanglement more or less the same. In particular, we point out that for $\gamma =3\Gamma /4$, the maximum value of entanglement for the symmetric bidirectional case shows a $15\%$ reduction compared to the $\gamma =0$ situation. Here we emphasize that the chirality not only assists in achieving elevated values of maximum entanglement in the presence of spontaneous emission but also helps to decrease somewhat the difference in the ${\mathscr {F}}_{\Delta , max}$ value (see for example, the most chiral situation of $\Gamma _r=5\Gamma _l$ in which the maximum entanglement difference reduces to $10\%$ compared to the corresponding $\gamma =0$ problem).

## Conclusions and outlook

In this paper, we studied the generation and control of three-photon Gaussian wavepacket-induced entanglement between two to three QEs side-coupled to chiral and symmetric bidirectional waveguides. Through the numerical solution of three-photon Fock state master equations, we calculated population dynamics and entanglement evolution which were quantified via bipartite concurrence and concurrence fill for two- and three-QE, cases respectively. At the single QE level, we found that chiral light-matter interaction was able to achieve $\sim 37\%$ maximum excitation percentage probability which is smaller than $\sim 52\%$ percentage probability obtained for the bidirectional symmetric coupling case. However, starting from 2 QE case chirality began to exhibit considerable improvement in both gaining higher entanglement values as well as single-QE excitation probability. More importantly, this trend extends down to the emitter-emitter entanglement where the bipartite concurrence reached maximum values that were five times larger than the symmetric case.

For the $N=3$ QE problem, we found that the chirality helps to sustain (at least) the single excitation probability (and hence the entanglement) for longer times. Furthermore, in the chiral case, we notice the phenomenon of tripartite entanglement death and revival. Importantly we point out that the maximum value achieved by the entanglement in all chiral cases (starting from $\Gamma _r=2\Gamma _l$ to $\Gamma _r=5\Gamma _l$) posed an upper bound on the maximum value of entanglement attained in the symmetric bidirectional problem ($\Gamma _r=\Gamma _l$). Compared to earlier studies of one and two-photon wQED where for two-qubit problem chirality is known to increase entanglement by a factor of 3/2 and 2, respectively; here for the three-photon case we have shown this enhancement to be $35\%$ (or by a factor of $\sim 5/14$). Finally, we discuss the impact of detuning and spontaneous emission on the generated tripartite entanglement. There we concluded both small detunings ($\omega _p-\omega _{eg}=\Gamma /2$) and spontaneous emission rate ($\gamma =3\Gamma /4$) retain the overall temporal profile of the entanglement. Detuning helps to sustain entanglement for longer times, while spontaneous emission rate results in a considerable reduction in the maximum value of entanglement. However, chirality still helped entanglement to show somewhat robustness against spontaneous emission loss.

The rich nature of the problem studied in this work allows the possibility of increasing the number of QEs and analyzing the impact of collective atomic effects on the generation of multipartite entanglement in the same setup. Also, we assumed in the present model that the inter-emitter separation was negligible. This assumption will be physically valid if the field’s wavelength is much larger than the atomic separation, which we inherently assume here. Note that these inter-emitter separations can also be presented in terms of the time retardation effect (the time it takes a photon to travel from one emitter to another as it propagates through the waveguide). There have been studies in the past where such effects have been ignored^54^ along with studies where such effects are taken into consideration (see Ref.^55^ where a wavefunction approach has been adopted). Because in our model, inter-emitter separations are negligible, consequently, such time retarded effects also don’t raise in our calculations. Examining the distance between emitters (or equivalently speaking the time retarded effects), which can lead to the potential non-Markovianity-induced revival of entanglement, could also be an exciting avenue of exploration. We leave these directions as the possible future extension of this work.

## Data Availability

The datasets used and/or analysed during the current study available from the corresponding author on reasonable request.
